# Effect of resistance training programs differing in set structure on muscular hypertrophy and performance in untrained young men

**DOI:** 10.3389/fphys.2023.1301535

**Published:** 2023-12-12

**Authors:** Jun Mao, Tianjiao Wang, Yidan Wang, Garry Kuan

**Affiliations:** ^1^ College of Kinesiology and Health, Capital University of Physical Education and Sports, Beijing, China; ^2^ Faculty of Sports and Exercise Science, Universiti Malaya, Kuala Lumpur, Malaysia; ^3^ Exercise and Sports Science Programme, School of Health Sciences, Universiti Sains Malaysia, Kubang Kerian, Kelantan, Malaysia

**Keywords:** resistance training, set structure, muscle hypertrophy, muscle performance, rest redistribution, cluster training

## Abstract

**Purpose:** This study aimed to compare the effects on muscle hypertrophy and muscular performance of two resistance training (RT) programs that differed only in set structure: traditional set structure (TS) vs. rest redistribution set structure (RR).

**Methods:** Thirty untrained young men were pair-matched and randomly assigned to a TS (*n* = 15) or an RR (*n* = 15) protocol based on individual baseline measures. Participants trained for 8 weeks using the same total body RT routines performed twice weekly. The TS protocol comprised four sets of 10 repetitions per exercise with 120-s interset rest, and the RR involved eight sets of five repetitions per exercise with 51-s interset rest. Participants were tested pre- and post-intervention for body composition, regional muscle thickness, upper- and lower-body muscle maximal strength [1-repetition maximum (1RM)], mean power output and velocity at 75% 1RM and muscular endurance (repetitions to failure at 70% 1RM).

**Results:** Compared to baseline, both groups exhibited equally significantly decreased body fat mass (*p* < 0.05), increased fat-free mass (*p* < 0.001), muscle thickness (*p* < 0.05), upper and lower-body muscular maximal strength (*p* < 0.001) and endurance performance (*p* < 0.001). However, both groups only increase the lower-body power output (*p* < 0.001) but not the upper-body (*p* > 0.05). No significant differences existed between groups for all measurements (*p* > 0.05).

**Conclusion:** These results suggest that RR and TS groups have similar effects for improving muscle hypertrophy and performance in untrained young men.

## 1 Introduction

Skeletal muscle mass and strength are crucial in physical function, athletic performance, and metabolic health. Resistance training (RT) has emerged as a powerful nonpharmacological method for increasing skeletal muscle mass and fitness (McKendry et al., 2011). The effectiveness of RT in promoting muscle hypertrophy and performance (strength, power, and endurance) relies on various factors within the training program, including volume, load intensity, rest intervals, frequency, time under tension, and type of contraction (American College of Sports Medicine, 2009). Traditionally, sets are performed without rest between repetitions, followed by a designated rest interval to allow recovery before proceeding to the next set. This conventional approach to RT set prescription is referred to as a traditional set structure (TS) (Tufano et al., 2017a).

During a traditional set of RT, individuals typically reach or near muscle failure, resulting in physiological and psychological fatigue. This fatigue is characterized by decreased concentric velocity and power performance (Izquierdo et al., 2006a), disruption of movement technique (Hooper et al., 2014), accumulation of metabolites (González-Hernández et al., 2020), and an increased effort perception (Lea et al., 2022). However, lower movement velocity and power in acute RT may hinder the maximization of long-term power development (Davies et al., 2017). In addition, although appropriate fatigue is essential for muscle hypertrophy and muscle performance gains, frequently reaching muscle failure can increase the risk of injury, and a higher perception of effort may discourage adherence to RT among the general public who are seeking to improve their health (Bibeau et al., 2010). One alternative approach to address these concerns is to use a cluster set structure (CS), which involves implementing short intra-rest (e.g., 10–45 s) periods within each set. Clear evidence suggests that in acute RT, CS is better than TS at maintaining concentric velocity and power and maintaining correct movement technique while eliciting lower metabolic stress and perceived effort (Hardee et al., 2013; Jukic et al., 2020). Moreover, CS confers benefits across diverse training statuses (Oliver et al., 2015), age (Dello Iacono et al., 2020), and gender (Boffey et al., 2021).

Previous research has established that CS can be categorized into two primary types. One is the basic cluster set structure (BCS), which refers to adding short breaks in a set (i.e., intra-set or inter-repetition rest interval) while maintaining regular rest periods between the two sets. However, BCS inevitably lengthens the total rest time compared to TS, which is a disadvantage for practitioners with limited exercise time. Another categorization known as rest redistribution (RR) redistributes the total rest time in TS to include shorter and more frequent intervals (Tufano et al., 2017a). The less total rest time may make RR less effective than BCS in reducing fatigue or maintaining movement performance in acute RT. However, it is a more time-efficient strategy and better applicable to real-world exercise settings (Jukic et al., 2020).

Although much research has been done on the acute advantages of RR (Tufano et al., 2017b; Boffey et al., 2021; Cuevas-Aburto et al., 2022), it is still uncertain what impact these acute effects will have on long-term muscle hypertrophy and performance adaptations. The existing body of evidence is limited and conflicting. Oliver et al. (2013) demonstrated that RR can produce better muscular strength and power gains while achieving similar fat-free mass gains as TS. In a similar experimental design study, Karsten et al. (2021) reported that RR was less effective in increasing muscle thickness but had better upper body power performance improvement. However, the two studies mentioned above did not strictly control for equal total rest time between the TS and RR groups, making the conclusions doubtful. In addition, the participants in the study by Oliver et al. (2013) and Karsten et al. (2021) were RT-experienced men, but it is unclear what chronic effects RR has on muscle hypertrophy and muscle performance in people with no RT experience.

From a practical point of view, understanding the similarities and differences between the effects of TS and RR on muscle hypertrophy and performance can yield valuable insights for the general population with the goal of promoting the musculoskeletal system. Since the RR set structure has the advantage of lower acute fatigue, it would be an attractive option if it had the same effect as TS on chronic muscle hypertrophy and muscle performance improvement. Consequently, the purpose of this study was to compare the effects of TS and RR on body composition, muscle thickness, muscular strength, power, and endurance of untrained young men using a moderate-intensity RT program with equal total training volume, intensity, and total rest time. Based on previous chronic studies on set structure (Jukic et al., 2021), we hypothesized: a) there will be no differences in body composition, muscle thickness, and maximal strength between groups, b) superior muscular power improvements for the RR, and c) superior muscular endurance improvements for the TS.

## 2 Materials and methods

### 2.1 Participants

*A priori* power analysis was performed using GPower (version 3.1.9.2) to calculate sample size. The input parameters used for the calculation were the following: F tests, ANOVA: Repeated measures, within-between interaction, two for the number of groups, two for the number of measurements, an alpha level of 0.05, a power (1-beta) equal to 0.8, and an effect size of 0.4 referenced to a previous similar experimental design (Karsten et al., 2021). The required minimum total sample size was estimated to be 16 (eight for each group), and the additional recruitment accounted for the possibility of dropouts.

A convenience sample of thirty-six physically active young male college students was initially recruited from the Capital University of Physical Education and Sports (Haidian District, Beijing, China). The inclusion criteria were: aged between 18 and 28 years, performing the physical activity at least twice a week (≥150 min moderate-intensity or 75 min vigorous-intensity/week) and not participating in resistance training in the past year. The exclusion criteria were any disease, injury, or other condition that could compromise the ability to perform the exercise. Thirty eligible participants were randomly allocated to one of the two intervention groups: TS (*n* = 15) and RR (*n* = 15). However, four participants (two from the TS group and two from the RR group) withdrew during the experiment for reasons unrelated to this study. Presented as mean ± SD the final groups characteristics were as follows: TS group: age: 21 ± 3 years, height: 180 ± 5 cm, and body mass: 79.2 ± 12.7 kg. RR group: age: 22 ± 3 years, height: 181 ± 9 cm, and body mass: 82.2 ± 16.6 kg. Before study initiation, the participants were fully informed of the study procedures and signed a written informed consent form. Participants were instructed not to change their physical activity and dietary habits, including participating in competitive sports or taking nutritional supplements.

### 2.2 Experimental design

**FIGURE 1:**
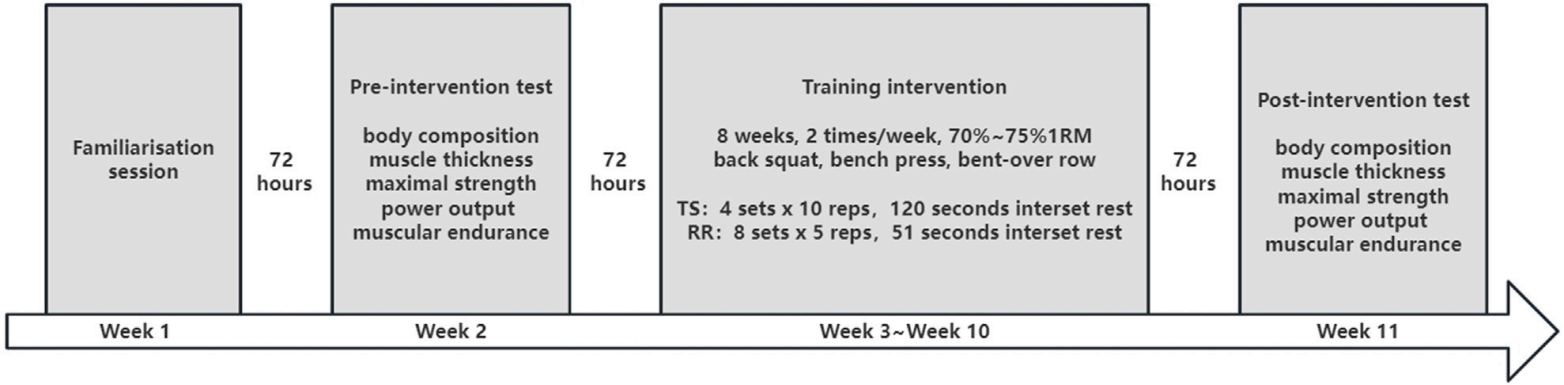
A schematic representation of the study.

The study used a two parallel group randomized controlled trial design. The whole study protocol comprised 11 weeks that included a familiarization session (week one), a pre-intervention test (week two), 16 training sessions (week three to week ten), and a post-intervention test (week eleven) (Figure 1). After the familiarization session and baseline testing, the participants were randomly assigned into the TS or RR groups and matched according to baseline physical and performance characteristics. Two sessions were performed each week, separated by 72 h during the training period. Before and after the intervention period, body composition, regional muscle thickness, strength, power, and endurance were assessed. Testing and training sessions were separated by at least 72 h. All muscle performance testing and training sessions were performed at a consistent time of the day (4:00 p.m. to 9:00 p.m.) for the individual participant. Ethical approval was granted by the Capital University of Physical Education and Sports Ethical Committee (2023A002).

### 2.3 Familiarization period

Before the start of the intervention, participants performed three familiarization sessions with a one-day rest between training sessions. Movement standards followed guidelines established by the National Strength and Conditioning Association (NSCA) (Haff and Triplett, 2015). The correct movements and testing procedures were explained and instructed during the familiarization training sessions by two experimenters who are NSCA-certified strength and conditioning specialists.

### 2.4 Test procedure

Participants performed tests during the week after a 1-week familiarization session and after all intervention sessions. Each participant took the test 72 h after the last familiarization and training sessions. Participants refrained from vigorous physical activity 72 h before all pre and post-intervention tests. Each participant came to the laboratory in the morning (7:00 a.m. to 11:00 a.m.) after a whole night of fasting (>10 h) and was measured in the following order: a) anthropometric, b) body composition and c) muscle thickness. Muscle performance testing is conducted over 3 days, each focusing on a specific movement: bench press, bent-over row, and back squat. These tests are carried out within a defined time frame from 4:00 p.m. to 9:00 p.m. with a 24–48 h interval between test sessions. The muscle performance tests of each movement were performed in the following order: a) maximum strength, b) power output, and c) muscular endurance. The bent-over row was not included in the muscle power test. There was a 10 minutes rest between each muscle performance test. Participants characteristics and baseline are presented in Table 1.

**TABLE 1:** Characteristics of participants at baseline.

Measure	TS (*n* = 13)	RR (*n* = 13)	*p*
Age (year)	21 ± 3	22 ± 3	0.765
Height (cm)	180 ± 5	181 ± 9	0.576
Body mass (kg)	79.2 ± 12.7	82.1 ± 16.6	0.617
Fat mass (kg)	15.1 ± 8.3	17 ± 8.4	0.569
Fat mass (%)	18.2 ± 7.2	19.8 ± 5.5	0.511
Fat-free mass (kg)	64.1 ± 5.8	65.2 ± 8.9	0.725
Fat-free mass (%)	81.8 ± 7.2	80.2 ± 5.5	0.511
Pectoralis major MT (cm)	2.02 ± 0.32	1.88 ± 0.38	0.323
Biceps brachii MT (cm)	2.26 ± 0.29	2.18 ± 0.30	0.481
Rectus femoris MT (cm)	2.48 ± 0.54	2.43 ± 0.33	0.763
BP 1RM (kg)	71 ± 13	69 ± 14	0.613
BP 1RM_BM_ (kg·kg^−1^)	0.9 ± 0.2	0.9 ± 0.3	0.598
BR 1RM (kg)	69 ± 8	67 ± 7	0.428
BR 1RM_BM_ (kg·kg^−1^)	0.9 ± 0.1	0.8 ± 0.2	0.408
BS 1RM (kg)	111 ± 16	115 ± 20	0.593
BS 1RM_BM_ (kg·kg^−1^)	1.4 ± 0.2	1.4 ± 0.3	0.780
BP mean power (W)	336 ± 77	338 ± 70	0.936
BS mean power (W)	669 ± 135	642 ± 192	0.679
BP mean velocity (m/s)	0.58 ± 0.14	0.62 ± 0.08	0.427
BS mean velocity (m/s)	0.74 ± 0.09	0.68 ± 0.12	0.177
BP endurance (repetitions)	15 ± 3	16 ± 3	0.814
BP endurance (volume, kg)	750 ± 150	764 ± 259	0.866
BR endurance (repetitions)	16 ± 2	15 ± 3	0.450
BR endurance (volume, kg)	771 ± 67	722 ± 157	0.308
BS endurance (repetitions)	17 ± 3	18 ± 3	0.292
BS endurance (volume, kg)	1334 ± 333	1477 ± 334	0.287

kg, kilogram; cm, centimetre; W, watt; m, metre; s, second; MT, muscle thickness; 1RM, one repetition maximum; BM, body mass; BP, bench press; BR, bent-over row; BS, back squat.

#### 2.4.1 Anthropometric

Participants’ height (cm) were measured in light clothing with shoes off using a regularly calibrated ultrasonic tester (DHM-200, China). Height were recorded to the nearest 0.5 cm.

#### 2.4.2 Body composition

The body composition, including body fat and fat-free mass, was assessed using a bioelectrical impedance analyzer (Tanita MC-980MA, Tokyo, Japan). Participants were tested for body composition under standardized conditions (early morning, overnight fasted, refrained from water intake, and empty bladder), wearing light clothes and no footwear. Participant age, sex and height were entered into the software; they then stood barefoot on the device to determine their body mass; once body mass had been determined, the participants grasped the handles of the device with both hands, keeping both arms alongside the body, and a complete segmental analysis was performed in less than 30 s. The body mass data measured by Tanita MC-980MA was used for analysis.

#### 2.4.3 Muscle thickness

**FIGURE 2:**
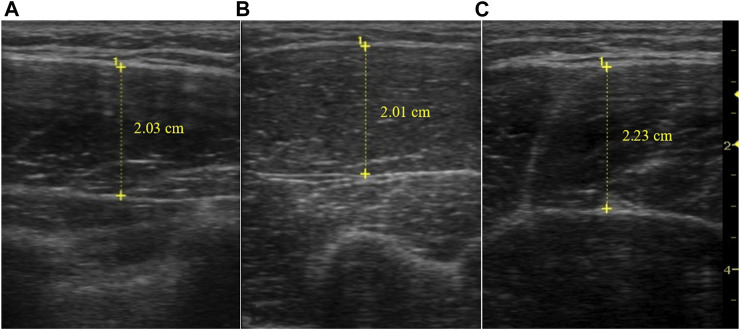
Example ultrasound images of the pectoralis major **(A)**, biceps brachialis **(B)**, and rectus femoris **(C)** muscle, measurement depth is 5 cm.

Muscle thickness of the pectoralis major, biceps brachii and rectus femoris was measured by a skilled technician using a portable B-mode ultrasound machine (LOGIQ Book XP, GE Healthcare, Wauwatosa, WI, United States) equipped with a 10-MHz linear probe. Each participant was placed at a treatment table in a relaxed position, with knees fully extended and arms held straight alongside the torso with a supination position of the lower arms. The measurement sites were accurately located and marked at the 60% distal to the lateral humerus epicondyle from the scapular acromial process for the biceps brachialis muscle and at the site between the third and fourth of costa under the clavicle midpoint for the pectoralis major muscle; and at the mid-point between the anterior superior iliac spine and the lower edge of the patella for the rectus femoris muscle. After a generous application of a water-soluble transmission gel to the site to be evaluated, a linear probe was placed perpendicular to the tissue interface without depressing the skin. Equipment settings were optimized for image quality, according to the manufacturer’s user manual and held constant among testing sessions. When the quality of the image was deemed to be satisfactory, the image was saved to the hard drive, and muscle thickness dimensions were obtained by measuring the distance from the subcutaneous adipose tissue–muscle interface to the muscle-bone interface. Images were obtained 72 h before and after the training intervention to avoid intramuscular swelling. Three images were obtained for each site in a single session, with the mean of the two closest values used for analysis. The reliability of muscle thickness measurements was measured by the same skilled technician on different days and was assessed using the intraclass correlation coefficient (ICC) with a two-way random model of absolute agreement. The ICC for measuring pectoralis major muscle thickness was 0.989 (Day one: 1.99 ± 0.28, Day two: 1.96 ± 0.30, CV = 2.6%, SEM = 0.05, MDC = 0.14), the biceps brachialis muscle thickness was 0.984 (Day one: 2.33 ± 0.30; Day two: 2.35 ± 0.29; CV = 2.3%, SEM = 0.05, MDC = 0.15), and the rectus femoris muscle thickness was 0.993 (Day one: 2.61 ± 0.58; Day two: 2.58 ± 0.59; CV = 2.1%, SEM = 0.06, MDC = 0.15). Example images of the muscle thickness are presented in Figure 2.

#### 2.4.4 Maximum strength

The maximum strength of the upper and lower-body was assessed by one repetition maximum (1 RM) of bench press, bent-over row and back squat using a barbell and plates on a power rack (Keiser, United States). Participants refrained from any exercise at least 48 h before pre- and post-intervention testing. The maximum strength testing process and movement standards were consistent with recognized guidelines established by the NSCA (Haff and Triplett, 2015) and supervised by an NSCA-certified strength and conditioning specialist. In brief, participants completed a general warm-up before testing, which consisted of 5 minutes of jogging and dynamic stretching lasting approximately 10 minutes. Dynamic stretches involve the chest, back, shoulder, abdominal, thigh, and gluteal muscle groups, with upper body stretches followed by lower-body stretches, with 5–10 repetitions of each movement or each side. Subsequently, a specific warm-up set of the given exercise of 8–10 repetitions was performed at 50% estimated 1RM, followed by two sets of 2–3 repetitions at a load corresponding to 60%–80% estimated 1RM. Participants then performed sets of one repetition with increasing weight for actual 1RM determination. Three to 5 minutes of rest was provided between each successive attempt. All 1RM determinations were made within five attempts.

#### 2.4.5 Power output

The mechanical power output of the upper and lower-body was assessed during the concentric phase of the bench press and back squat exercises using a relative load of 75% of pre- or post-intervention 1RM. The power test is performed 10 minutes after the maximal strength test. Power output was measured using an inertial sensor (Vmaxpro, Blaumann and Meyer—Sports Technology UG, Magdeburg, Germany) attached to the middle of the barbell bar. The validity and reliability of Vmaxpro have been previously reported (Feuerbacher et al., 2022; Haynes, 2022). Participants were required to perform three repetitions with a correct form and perform the concentric phase of each lift with the maximal possible movement velocity. If the power output of the third repetition exceeded the first two, participants completed two more repetitions until the power output declined. If the power output increased over five repetitions, they took a 3 minutes break and retested until the highest average power was achieved. The highest average power output was used for statistical analysis.

#### 2.4.6 Muscular endurance

Muscular endurance of the upper and lower-bodys was evaluated by the maximal number of repetitions for the bench press, bent-over row and back squat exercises with 70% of pre- or post-intervention 1RM. The muscular endurance is performed 10 min after the power output test. Participants were required to perform the movement with a correct form and with the maximal possible concentric movement velocity until muscular exhaustion, defined as when the weight ceased to move, or the participants failed to maintain the correct technique. Any repetition without a full range of movement was not counted. The total number of repetitions completed and volume (repetitions × load) were used for analysis.

### 2.5 Training intervention protocol

The RT program in this study was designed with muscle hypertrophy as the primary objective and comprised three upper- and lower-limb exercises performed in the following order during each training session: back squat, bench press and bent-over row. Participants were randomly allocated to TS (*n* = 15) or RR (*n* = 15) intervention groups and completed 16 training sessions over eight consecutive weeks, two sessions per week with a minimum of 72 h between sessions. Except for four participants who dropped out of the experiment, the remaining participants (*n* = 26) completed all training sessions. The two intervention groups were equalized in volume, intensity, frequency and total rest time but differentiated in the set structure, including different numbers of sets and interset rest. The TS protocol comprised 4 sets of 10 repetitions per exercise with 120-s rest between sets, and the RR comprised 8 sets of 5 repetitions per exercise with 51-s rest between sets. There would be a 5 minutes interval between each movement, and participants would rest for two to 3 minutes after completing the previous movement, then complete a warm-up set (five to ten reps) of the next movement at 50% of the target weight (e.g., half the weight of a 70% 1RM), followed by another two to 3 minutes of rest before beginning the formal set. The same warm-up set will be done before the first movement (back squat). The experimental supervisor controlled the rest time by using the countdown function of the smartphone. The requirements for movement techniques in the RT intervention session were the same as during the muscle performance test. The participants were instructed to perform the concentric phase of all lifts as fast as possible and to lower the barbell in a controlled manner in 2 seconds. The experimental supervisor helped participants control the eccentric tempo of the movements by playing sounds (30 beats per minute) using metronome software on the smartphone with an external speaker. When completing all workouts, participants were asked to focus on movement technique and tempo rather than a particular muscle or muscle group. In the TS group, participants did not reach muscle failure on every set. However, they gradually accumulated fatigue, reaching or approaching muscle failure as the number of sets completed increased, as evidenced by an inability to complete the concentric portion of the movement. Verbal encouragement was provided throughout training. If the participant was unable to complete the prescribed number of repetitions in a set, the load was reduced by 10% in the subsequent set. The RT programs increased load intensity over time, with a load intensity of 70% 1RM in week three, 72.5% 1RM in week four, and 75% 1RM in weeks five and six. 1RM was remeasured in week six, 72 h after the final training session; the load intensity was 70% 1RM in week seven, 72.5% 1RM in week eight, and 75% 1RM in weeks nine and ten. According to relevant RT guidelines (American College of Sports Medicine, 2009) and previous research (Schoenfeld et al., 2017; Androulakis-Korakakis et al., 2020; Fyfe et al., 2022), the load intensity, training duration and training volume pre week used in the TS program in our study were sufficient to elicit measurable increases in muscle mass and strength among untrained young males. Participants began their first training session in week three, 2 days after completing the pre-intervention test, and the post-intervention test was performed 2 days after the last training session in week ten.

#### 2.5.1 Statistical analyses

A descriptive analysis was performed, and subsequently, the Kolmogorov-Smirnov and Shapiro-Francia tests were applied to assess normality. All pre- and post-intervention data were summarized and reported as mean (SD). The reliability of muscle thickness measurements was calculated using intraclass correlation coefficients (ICCs). The results of the ICCs were interpreted as <0.5 poor, 0.5 to 0.75 moderate, 0.75 to 0.9 good, and >0.9 excellent reliability (Koo and Li, 2016). The independent samples *t*-test was used to establish the difference between the baseline variables and the difference of change values between the groups. Changes in all outcome variables were calculated by subtracting pre-from post-test values. Primary outcomes were analyzed using a 2 × 2 (group by time) repeated-measures analysis of variance with a *post hoc* Bonferroni adjustment multiple comparisons. The interpretation of the effect size: small, ηp^2^ = 0.01; medium, ηp^2^ = 0.06; and large, ηp^2^ = 0.14. Cohen’s d was used to measure between-group effect size, with a small, medium, and large effect equal to 0.2, 0.5, and 0.8, respectively (Cohen, 1992). For between-group effects, positive effect sizes indicate that the effect favored TS, whereas negative effects favored RR. An alpha level of significance was set at *p* < 0.05 for all analyses.

## 3 Results

### 3.1 Body composition

Data for measures of body composition are presented in Table 2. There were significant time effect but no significant group or group by time interactions (*p* > 0.05) for body fat mass, body fat percentage, fat-free mass and fat-free mass percentage (*p* > 0.05). Compared to baseline, both groups significantly decreased body fat mass (F = 18.013, *p* < 0.001, ηp^2^ = 0.429) and body fat percentage (F = 19.665, *p* < 0.001, ηp^2^ = 0.45). Compared to baseline, both groups significantly increased fat-free mass (F = 12.894, *p* = 0.001, ηp^2^ = 0.349) and fat-free mass percentage (F = 19.665, *p* < 0.001, ηp^2^ = 0.45).

**TABLE 2:** Changes in body composition, regional muscle thickness, muscular strength, power output and endurance pre- and post-the intervention period.

Measure	TS (*n* = 13)		RR (*n* = 13)		ANOVA (*p*)			Between-group	
	Pre	Post	Pre	Post	G	T	G × T	*P*	ES
Body mass (kg)	79.2 ± 12.7	78.7 ± 11.8	82.1 ± 16.6	81.2 ± 15.8	0.636	0.131	0.596	0.596	0.21
Fat mass (kg)	15.1 ± 8.3	13.7 ± 7.2	17 ± 8.4	14.9 ± 7	0.613	< 0.001#	0.398	0.398	0.34
Fat mass (%)	18.2 ± 7.2	16.7 ± 6.2	19.8 ± 5.5	17.7 ± 4.2	0.557	< 0.001#	0.430	0.416	0.33
Fat-free mass (kg)	64.1 ± 5.8	65 ± 5.4	65.2 ± 8.9	66.3 ± 9.1	0.698	0.001#	0.714	0.714	−0.15
Fat-free mass (%)	81.8 ± 7.2	83.3 ± 6.2	80.2 ± 5.5	82.3 ± 4.2	0.557	< 0.001#	0.430	0.416	−0.33
Pectoralis major MT (cm)	2.02 ± 0.32	2.17 ± 0.37	1.88 ± 0.38	2.03 ± 0.37	0.316	0.001#	0.992	0.992	0.00
Biceps brachii MT (cm)	2.26 ± 0.29	2.34 ± 0.30	2.18 ± 0.30	2.25 ± 0.22	0.393	0.043#	0.817	0.817	0.09
Rectus femoris MT (cm)	2.48 ± 0.54	2.72 ± 0.47	2.43 ± 0.33	2.63 ± 0.27	0.655	< 0.001#	0.709	0.709	0.15
BP 1RM (kg)	71 ± 13	84 ± 15	69 ± 14	81 ± 16	0.637	< 0.001#	0.946	0.946	−0.03
BR 1RM (kg)	69 ± 8	82 ± 7	67 ± 7	80 ± 7	0.454	< 0.001#	0.793	0.793	−0.10
BS 1RM (kg)	111 ± 16	143 ± 23	115 ± 20	145 ± 16	0.677	< 0.001#	0.733	0.733	0.14
BP mean power W)	336 ± 77	368 ± 87	338 ± 70	355 ± 66	0.827	0.119	0.608	0.608	0.20
BS mean power W)	669 ± 135	811 ± 175	642 ± 192	806 ± 154	0.791	< 0.001#	0.673	0.673	−0.17
BP mean velocity (m·s^-1^)	0.58 ± 0.14	0.56 ± 0.09	0.62 ± 0.08	0.57 ± 0.10	0.505	0.226	0.569	0.569	0.23
BS mean velocity (m·s^-1^)	0.74 ± 0.09	0.70 ± 0.08	0.68 ± 0.12	0.67 ± 0.08	0.172	0.237	0.465	0.465	−0.29
BP endurance (repetitions)	15 ± 3	15 ± 2	16 ± 3	16 ± 2	0.462	0.910	0.653	0.653	−0.18
BP endurance (volume, kg)	750 ± 149	881 ± 223	764 ± 259	906 ± 228	0.810	< 0.001#	0.874	0.867	−0.07
BR endurance (repetitions)	16 ± 2	16 ± 2	15 ± 3	16 ± 3	0.453	0.404	0.867	0.132	0.61
BR endurance (volume, kg)	771 ± 67	939 ± 111	722 ± 157	897 ± 189	0.353	< 0.001#	0.894	0.874	−0.06
BS endurance (repetitions)	17 ± 3	18 ± 4	18 ± 3	17 ± 2	0.720	0.700	0.132	0.894	−0.05
BS endurance (volume, kg)	1334 ± 333	1773 ± 410	1477 ± 334	1764 ± 357	0.595	< 0.001#	0.270	0.270	0.44

kg, kilogram; cm, centimetre; W, watt; m, metre; s, second; MT, muscle thickness; 1RM, one repetition maximum; BP, bench press; BR, bent-over row; BS, back squat; T, time; G = group; G × T, group by time interaction; #, significant (*p* < 0.05).

### 3.2 Regional muscle thickness

Data for measures of regional muscle thickness are presented in Table 2. There were significant time effect but no significant group or group by time interactions (*p* > 0.05) for pectoralis major, biceps brachii and rectus femoris muscle thickness. Compared to baseline, both groups significantly increased pectoralis major (F = 13.5, *p* = 0.001, ηp^2^ = 0.36), biceps brachii (F = 4.550, *p* = 0.043, ηp^2^ = 0.159), and rectus femoris (F = 22.247, *p* < 0.001, ηp^2^ = 0.481) muscle thickness.

### 3.3 Muscular strength

Data for measures of muscle maximal strength are presented in Table 2. There were significant time effect but no significant group or group by time interactions (*p* > 0.05) for bench press, bent-over row and back squat 1RM. Compared to baseline, both groups significantly increased bench press 1RM (F = 78.005, *p* < 0.001, ηp^2^ = 0.765), bent-over row 1RM (F = 151.736, *p* < 0.001, ηp^2^ = 0.863) and back squat 1RM (F = 126.645, *p* < 0.001, ηp^2^ = 0.841).

### 3.4 Power output and velocity

Data for measures of muscle mean power output and velocity are presented in Table 2. There were significant time effect but no significant group or group by time interactions (*p* > 0.05) for back squat mean power. Compared to baseline, both groups significantly increased back squat mean power (F = 32.733, *p* < 0.001, ηp^2^ = 0.577).

### 3.5 Muscular endurance

Data for measures of muscular endurance are presented in Table 2. There were significant time effect but no significant group or group by time interactions (*p* > 0.05) for bench press, bent-over row and back squat endurance volume. Compared to baseline, both groups significantly increased bench press (F = 17.431, *p* < 0.001, ηp^2^ = 0.421), bent-over row (F = 47.717, *p* < 0.001, ηp^2^ = 0.665), and back squat endurance volume (F = 29.458, *p* < 0.001, ηp^2^ = 0.551).

## 4 Discussion

The main finding of the current study was that both TS and RR groups resulted in a similar increase in fat-free mass, muscle thickness, and decreased body fat. Regarding muscle performance, both groups showed significant increases in upper-body and lower-body maximum strength and endurance, but only the lower-body power was improved. Contrary to our hypothesis, the RR and TS groups did not demonstrate differences in muscular power and endurance adaptations.

Our results found that both the TS and RR groups increased fat-free mass and muscle thickness. Typical muscle hypertrophy training involves moderate loading intensity ranging from 60% to 80% 1RM, 8 to 12 repetitions per set, and performing repetitions at or near muscle failure. This type of training can disrupt cellular homeostasis and generate higher metabolic stress. Metabolic stress is manifested as a result of the exercise-induced accumulation of metabolites such as lactate, phosphate inorganic (Pi), and ions of hydrogen (H^+^) in muscle cells, which is essential for muscle growth (Schoenfeld, 2013; de Freitas et al., 2017). Previous findings suggest TS can cause higher metabolite accumulation than RR (Jukic et al., 2020). It has been suggested that higher metabolic stress promotes more anabolic hormones, which is favorable to muscle anabolism (Schoenfeld, 2013). However, existing studies all point to BCS and RR eliciting lower blood lactate but a similar endocrine response to TS when training volume and total rest time are matched (Girman et al., 2014; Oliver et al., 2015; Merrigan et al., 2020). Moreover, whether the additional metabolic stress generated by muscle failure in each set leads to greater muscle gain is still being determined. There may be a threshold for metabolic stress above which no further beneficial effects are observed (Schoenfeld and Grgic, 2019).

In contrast to our results, Karsten et al. (2021) found that the TS group (repetition-to-failure per set) had superior hypertrophic outcomes compared to the RR group (not-to-failure per set). The difference in results may be attributed to variations in the implementation of RT between the two studies. In the study by Karsten et al., participants in the TS group were instructed to use a self-estimated 10RM load to ensure that muscle failure was achieved in each set of exercises. On the other hand, the RR group self-selected load intensity and adjusted it based on the OMNI-resistance exercise scale to maintain a final perceptual value not exceeding seven at the end of each set. However, this method of load intensity adjustment could potentially result in a disparity in the actual training volume between the two groups. Furthermore, since the TS group performed exercises until muscle failure in each set, it is possible that other factors, such as increased mechanical tension (Schoenfeld, 2010), greater muscle fibre activation (Morton et al., 2019), and more muscle damage (Shibata et al., 2021), contributed to the observed more significant muscle growth in the TS group. However, the observed changes in muscle hypertrophy in our study are consistent with the findings of Oliver et al. (2013), even though the participants in their research had RT experience and followed a high-volume program. It is worth noting that our study and Oliver et al. (2013) utilized a percentage of one repetition maximum (% 1RM) approach to determine RT loads and match training volume. Therefore, it can be inferred that when the training intensity and volume are equal, and the TS group is not obligated to reach momentary muscle failure on every set, the RR method leads to comparable hypertrophic adaptations as TS, irrespective of the individual’s training status.

Consistent with previous studies, our research also found that RR and TS have similar effects on maximal dynamic strength (Davies et al., 2021; Jukic et al., 2021). According to Henneman’s size principle (Henneman et al., 1965), lower threshold motor units are initially recruited to lift weights during resistance exercise sets using moderate loads. As these lower threshold motor units become fatigued, higher threshold motor units are recruited to maintain force production. Therefore, it is hypothesized that performing RT sets until reaching or near momentary muscle failure would activate more motor units and ultimately maximize muscle strength (Fisher et al., 2011). However, recent studies have not supported that reaching muscle failure in RT provides additional benefits for strength gains (Grgic et al., 2022). In fact, frequent muscle failure can lead to overtraining and overreaching (Fry and Kraemer, 1997; Schoenfeld and Grgic, 2019), which are detrimental to strength development. Surprisingly, our findings are inconsistent with Oliver et al. (2013) despite using a similar set structure and loading in our RT program design. The discrepancies may be attributed to differences in other experimental variables, such as participants’ training status, the training volume per session, and the training frequency. In the study of Oliver et al. (2013), the participants performed seven exercises per session, four sessions per week, whereas in our study, participants performed three exercises per session, two sessions per week. Individuals with prior resistance training experience may require higher volume and frequency of training to obtain the benefits of RR in increasing maximal strength. It is worth noting that although Oliver et al. (2013) observed that RR resulted in better strength and power improvements compared to TS, muscle biopsy results indicated similar increases in MHC IIA percentage and decreases in MHC IIx percentage in both the RR and TS groups, which suggests that the enhanced muscle performance achieved through RR may primarily be attributed to neural rather than morphological factors (Cormie et al., 2011).

Contrary to our research hypothesis, despite observing a lower-body power increase in both groups, the RR group did not exhibit any advantage in power improvement compared to the TS group. Several potential reasons could explain this phenomenon. Firstly, participants in this study were instructed to perform the concentric portion of the exercise as fast as possible, whereas performing the movement at the maximum intended concentric velocity (with an actual lower velocity) may yield similar results as performing at a high movement concentric velocity (Kawamori and Newton, 2006). Second, although it has been shown that faster acute concentric velocity in RT is beneficial for enhancing muscular power (Pareja-Blanco et al., 2014), we speculate that the difference in acute concentric performance between the two set structure protocols in the present study may not be sufficient to elicit significant differences in velocity and power adaptations after 8 weeks of training or 16 training sessions. Our study also observed no increase in the average velocity of the 75% 1RM bench press and back squat in either the TS or RR group and even a slight decrease in general. Considering the force-velocity and force-power relationships for concentric contractions of skeletal muscle, it is known that an increase in force output at a constant velocity leads to an increase in power (Cormie et al., 2011). Therefore, it can be inferred that the increase in lower muscle power primarily stems from the gain in maximal strength (Taber et al., 2016). The reason for the lack of significant power gains in the bench press in this study may also be related to the insufficient maximal strength gains in the bench press.

Additionally, according to the velocity specificity principle (Kawamori and Newton, 2006), the test results are influenced by the specificity of the test method, including the movement and load intensity. Therefore, we conducted a power test using the same movements and submaximal intensities as the RT program. In contrast to our study, Oliver et al. (2013) found that RR produced better power output performance (60% 1RM bench press and squat, vertical jump) gains than TS. Karsten et al. (2021) also reported that RR produced better upper body power (50% 1RM bench press) adaptations than TS. One possible explanation for the inconsistent results between studies could be the significant difference in relative intensities used for power testing. Oliver et al. (2013) and Karsten et al. (2021) utilized much lower relative load intensities than our research. Therefore, it can be suggested that RR-induced better power adaptation may only occur when relative intensities are below 75% 1RM. However, further research is needed to confirm this hypothesis.

Considering the higher velocity loss and closer proximity to muscle failure in TS compared to RR (Jukic et al., 2020), we hypothesized that TS would lead to superior improvements in muscular endurance. However, the present study discovered that the TS and RR groups demonstrated equal effects in muscular endurance for both the upper and lower-body. A study conducted by Izquierdo et al. (2006a) examined training-to-failure *versus* non-failure (which completed half the number of maximal repetitions) in RT. They found that the training-to-failure group showed more significant improvements in upper-body muscular endurance than the non-failure group, with no significant difference in the lower-body. It is important to note that Izquierdo et al. (2006b) did not match the total rest periods, resulting in the non-failure group having more recovery time than the training-to-failure group. This difference in recovery time may have led to distinct physiological responses and adaptations between the two groups (Gorostiaga et al., 2012). However, the focus of this study was on set structure, the total rest time for TS and RR was equated, and the TS protocol in this study did not require each set to perform to muscular failure, which may have narrowed the difference in physiological demands between the two set structure in terms of muscle resistance to fatigue. In addition, the testing method also affects the results; Izquierdo et al. (2006a) used absolute loads (75% 1RM in the pre-test), whereas our study used relative loads (70% 1RM) for localized muscular endurance testing. Consequently, similar maximal strength gains might have contributed to similar muscular endurance gains in both groups. Another study conducted a muscular endurance test using an absolute load (10RM in pre-test) found that the TS group demonstrated increased biceps muscular endurance, while the RR group did not show the same improvement (Fariñas et al., 2021). However, the studies mentioned above utilized an inter-repetition rest set structure and single-joint exercises, which differs from ours. It is worth noting that RR may perform better in other types of endurance tests. For instance, Janicijevic et al. (2022) conducted a muscular endurance test using a two set structure (TS and RR) before and after the intervention for the TS and RR groups, respectively. The participants completed ten repetitions of the bench press or back squat with a load of approximately 75% of 1RM. The results indicated that the RR group achieved greater gains in the mean velocity of the bench press, regardless of the set structure used for testing. Future studies should consider simultaneously conducting multiple muscular endurance tests to explore the effect of set structure on muscle performance.

This study has several limitations that must be considered when attempting to draw evidence-based inferences. First, only physically active young men with no RT experience were included in this study, so the findings cannot be extrapolated to other populations (e.g., females, adolescents, and older adults) and the RT-experienced population. Second, the present study consisted of only three multi-joint movements with a relatively low training volume. However, RT programs aimed at muscle hypertrophy should include a greater variety of exercises and higher volume to stimulate muscle growth fully (Schoenfeld et al., 2017). Future studies should attempt to include more movements and high training volume. In addition, the duration of the training intervention in this study was relatively short. It may require a longer time for differences in physiological adaptations induced by RT in different set structures to become apparent, especially considering the lack of RT experience among the participants in this study. For body composition measurements, using the bioelectrical impedance analysis is more influenced by hydration status and may be less accurate than air displacement plethysmography and dual-energy x-ray absorptiometry measurements (Lemos and Gallagher, 2017). For the power test, the inertial sensors used in this study to measure movement velocity and power may also be less valid than multiple linear position transducers synchronized with a force plate. Moreover, the power test load intensity used in this study was 75% 1RM, but the load intensity to produce maximum power output was much lower for the bench press and back squat movements; further research is needed to determine the advantage of rest redistribution in improving maximal power. Lastly, acute fatigue and performance of the TS and RR protocols were not measured in this study, and these data are useful for analyzing and interpreting the characteristics and application scenarios of the different set structures.

## 5 Conclusion

In summary, the findings of this study indicate that 8 weeks of RT programs using both TS and RR set structure effectively increased fat-free mass, muscle thickness, muscular strength, power, and endurance in untrained young men. However, there was no significant difference in the adaptation induced by TS and RR.

## Data Availability

The original contributions presented in the study are included in the article/supplementary materials, further inquiries can be directed to the corresponding author.

## Abbreviations

RT, Resistance training; TS, Traditional set structure; CS, Cluster set structure; BCS, Basic cluster set structure; RR, Rest redistribution; BM, Body mass; MT, Muscle thickness; RM, Repetition maximum; BP, Bench press; BR, Bent-over row; BS, Back squat.
